# Global surveillance of *Candida albicans*: MIC distributions, resistance patterns, and temporal trends from the Antimicrobial Testing Leadership and Surveillance program (2015–2024)

**DOI:** 10.1128/aac.01617-25

**Published:** 2026-05-12

**Authors:** Guiyue Cai, Zhanpeng Zhang

**Affiliations:** 1 Department of Dermatology, The First Affiliated Hospital of Jinan University 162698, Guangzhou, China; University Children's Hospital Münster, Münster, Germany

**Keywords:** antifungal resistance, minimum inhibitory concentration (MIC), echinocandins, azoles, global surveillance

## Abstract

*Candida albicans* remains the predominant cause of invasive candidiasis worldwide, yet comprehensive global analyses of its antifungal susceptibility trends remain scarce. In this decade-long study, we evaluated 6,342 *C. albicans* isolates collected between 2015 and 2024 through the Antimicrobial Testing Leadership and Surveillance global surveillance program, representing 41 countries across four continents. Europe accounted for more than half of all isolates, followed by North America, Asia–Western Pacific, and Latin America. The majority of isolates were derived from elderly and critically ill patients, particularly those hospitalized in internal medicine or intensive care units. Echinocandins and amphotericin B demonstrated sustained potency, maintaining low minimum inhibitory concentration (MIC)_50_/MIC_90_ values (0.015−0.03 mg/L and 0.5/1 mg/L, respectively) and ≥99% susceptibility throughout the surveillance period. In contrast, fluconazole and voriconazole exhibited broader MIC distributions and a subtle upward shift in MIC_90_ after 2022. Although global resistance rates remained below 1%, small numbers of resistant isolates were detected in several countries, including Greece (2.1%, *n* = 1), Belgium (1.1%, *n* = 4), Ireland (0.7%, *n* = 2), Turkey (0.5%, *n* = 2) and the United States (0.3%, *n* = 10). Temporal analysis further revealed a gradual increase in azole non–wild-type isolates from 2019 to 2024, while echinocandin susceptibility remained stable. These findings confirm that *C. albicans* remains broadly susceptible to frontline antifungal agents, yet the observed low-magnitude shifts and localized increases underscore the value of continued standardized surveillance and cautious, region-informed stewardship.

## INTRODUCTION

Invasive candidiasis represents a major cause of morbidity and mortality among hospitalized and immunocompromised patients, with *Candida albicans* consistently identified as the leading pathogen worldwide (1–3). Despite advances in antifungal therapy, candidemia and other invasive infections are frequently associated with high fatality—often exceeding 20%–30% in high-risk populations (4, 5).

Effective management of invasive *Candida* infections depends heavily on timely initiation of appropriate antifungal therapy. Echinocandins and amphotericin B are generally considered reliable first-line agents, while triazoles continue to play an important role in step-down treatment and prophylaxis (6, 7). However, widespread azole use has raised concerns about the development of resistance. Recent reports describe emergent increases in fluconazole minimum inhibitory concentrations (MICs) and clinically significant azole tolerance in *C. albicans* (8–10). Although global resistance rates in *C. albicans* remain relatively low compared with non-*albicans* species (11), incremental shifts in MIC distributions or the emergence of localized resistance patterns may warrant careful interpretation in the context of ongoing surveillance rather than immediate changes in clinical practice (3, 12).

Previous surveillance efforts, such as SENTRY and regional epidemiological programs, have provided valuable insight into antifungal susceptibility trends, but most have been limited by geographic scope, modest sample sizes, or short time span, thereby constraining their ability to detect subtle temporal dynamics or nascent resistance clusters (12–14). Comprehensive, longitudinal, and globally representative data are therefore essential to guide empirical therapy, inform antifungal stewardship, and support evidence-based monitoring of antifungal susceptibility over time (15).

To address these gaps, we evaluated over 6,300 *C. albicans* isolates collected between 2015 and 2024 through the Antimicrobial Testing Leadership and Surveillance (ATLAS) global surveillance program. We examined geographic distribution, minimum inhibitory concentration (MIC) distributions, susceptibility profiles, and temporal resistance dynamics across multiple regions. This study documents sustained antifungal activity alongside low-magnitude variability in azole susceptibility, reinforcing the importance of standardized, long-term surveillance to appropriately contextualize subtle changes in antifungal susceptibility over time.

## MATERIALS AND METHODS

### Study design and isolate collection

This study analyzed *Candida albicans* isolates collected between 2015 and 2024 through the ATLAS global surveillance program. The ATLAS protocol, initiated in 2006 by Pfizer (New York, NY, USA), systematically collects *in vitro* susceptibility data for clinically relevant bacterial and fungal pathogens worldwide using centralized testing, standardized methodologies, and uniform quality control procedures. The ATLAS surveillance framework builds upon long-standing global surveillance infrastructure, and portions of its historical isolate collections overlap with data sets generated through the SENTRY Antifungal Surveillance Program, which has been extensively described elsewhere. Relevant SENTRY publications are cited to provide continuity and transparency with prior global surveillance efforts.

Following the standardized ATLAS framework, only the first isolate per patient per infection episode per year was included to prevent duplication and overrepresentation of recurrent isolates. Clinical isolates were obtained from hospitalized patients across 41 participating countries spanning 4 continents (Europe, North America, Asia–Western Pacific, and Latin America). Specimen sources included blood, urine, respiratory tract secretions, wound exudates, peritoneal fluid, and other sterile sites from both intensive care and general wards. No additional patient-level clinical or treatment data (including prior antifungal exposure or clinical outcomes) were available for analysis. All isolates were collected with appropriate institutional ethical approval under local regulations.

### Species identification

Isolates were initially identified at local laboratories using conventional microbiological and biochemical methods. Final confirmation to the species level was performed centrally at JMI laboratory (North Liberty, Iowa, USA) using matrix-assisted laser desorption ionization time-of-flight mass spectrometry (MALDI-TOF MS) (Bruker Biotyper, Bruker Daltonics, Billerica, MA, USA).

### Antifungal susceptibility testing

MICs were determined using the broth microdilution method following Clinical and Laboratory Standards Institute (CLSI) guidelines (document M27M44S-ED3, CLSI 2022). The antifungal agents tested included the triazoles (fluconazole, voriconazole, itraconazole, posaconazole, and isavuconazole), echinocandins (caspofungin, micafungin, and anidulafungin), and amphotericin B.

Drug concentration ranges tested were 0.001–512 mg/L. Quality control was ensured using *Candida parapsilosis* ATCC 22019 and *Candida krusei* ATCC 6258 reference strains. Susceptibility interpretations followed the CLSI 2022 breakpoints for *C. albicans*. Wild-type (WT) and non–wild-type (NWT) classifications were determined strictly on the basis of CLSI epidemiologic cutoff values (ECVs). Isolates with MICs above the ECVs were designated as NWT.

### Data processing and analysis

All MIC data were curated through the ATLAS database (Pfizer Global Surveillance Platform). For each antifungal agent, MIC_50_, MIC_90_, and frequency distributions were calculated annually. Temporal changes in MIC distributions and WT/NWT proportions were assessed descriptively. No formal statistical trend analyses, regression modeling, or time-series testing was performed. Importantly, this study was not designed to determine whether observed MIC shifts exceeded the inherent variability of the broth microdilution assay, and modest shifts may reflect methodological variation rather than biologically or clinically meaningful resistance evolution.

Resistance rates were defined as the proportion of isolates with MIC values exceeding the CLSI clinical breakpoints. Both absolute isolate counts and corresponding percentages are reported to avoid overinterpretation of resistance frequencies derived from small denominators. Geographic mapping of isolate counts and resistance rates was performed using aggregated regional data sets (Europe, North America, Asia–Western Pacific, and Latin America). Only countries with ≥10 isolates were included in the resistance frequency analysis to ensure statistical robustness.

## RESULTS

### Global distribution of *C. albicans* isolates

**Fig 1:**
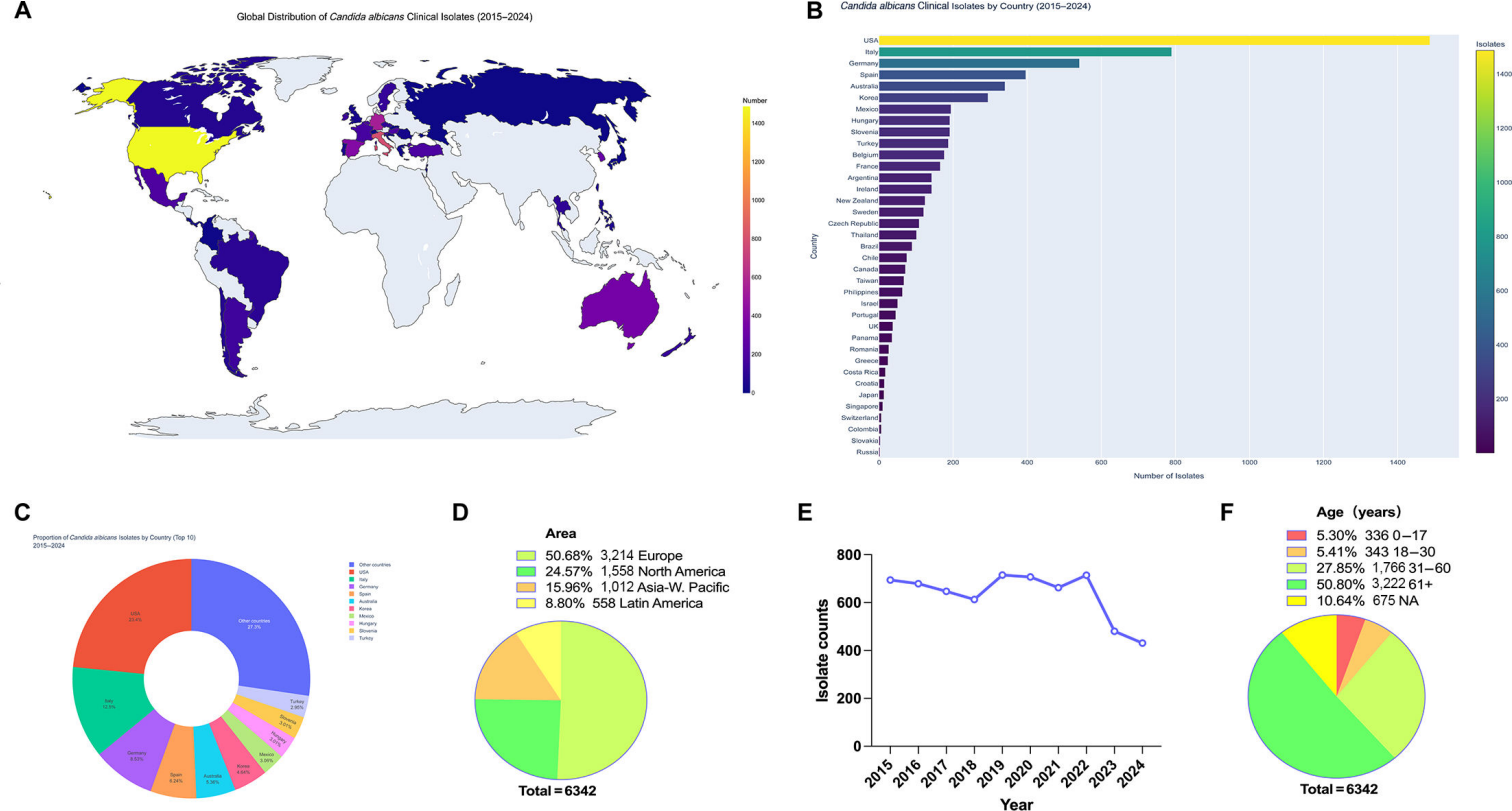
Global distribution and demographic characteristics of *Candida albicans* clinical isolates (2015–2024). Global distribution map (**A**), country-level isolate counts (**B**), top 10 national proportions (**C**), and continental proportions (**D**) illustrate the geographic scope of the ATLAS surveillance data set. Annual isolate trends (**E**) and patient age distribution (**F**) depict temporal and demographic characteristics of *C. albicans* isolates included in the analysis.

Between 2015 and 2024, a total of 6,342 *C. albicans* isolates were collected from 41 countries across four continents in the ATLAS surveillance program, providing a large, globally representative surveillance data set on antifungal susceptibility. Europe accounted for over half of all isolates (50.7%), followed by North America (24.6%), Asia–Western Pacific (16.0%), and Latin America (8.8%). Country-level distributions were dominated by the United States (23.5%), Italy (12.5%), and Germany (8.5%), reflecting strong contributions from Western surveillance networks (Fig. 1A through D). Annual isolate numbers remained relatively stable from 2015 through 2022 (approximately 650–715 per year) but declined sharply thereafter (480 in 2023 and 431 in 2024), suggesting possible pandemic-related or logistical impacts on sampling intensity (Fig. 1E).

Demographically, infections were heavily skewed toward older adults: 50.8% of isolates were derived from patients aged ≥61 years, compared with only 5.3% and 5.4% from pediatric and young adult populations, respectively (Fig. 1F). By clinical setting, the majority originated from internal medicine (20.4%) and intensive care units (16.9%), with notable representation from surgical wards (10.7%) and oncology/hematology units (5.8%), consistent with the recognized burden of *C. albicans* infections in hospitalized and immunocompromised populations (Table 1).

**TABLE 1:** Ward source of *Candida albicans* isolates (2015–2024)

Ward	Frequency	Percentage of total
Internal Medicine	1,293	20.39
Intensive Care Unit	1,072	16.9
Unknown	743	11.72
Surgery	679	10.71
Cardiothoracic/Pulmonary	433	6.83
Hematology/Oncology	370	5.83
Emergency	235	3.71
Pediatrics/Neonate	233	3.67
Ambulatory/Outpatient	157	2.48
Infectious Disease	154	2.43
Neurology	107	1.69
General/GI	105	1.66
Renal	98	1.55
Gastroenterology/GI	94	1.48
Obstetrics/Gynecology	73	1.15
Geriatrics	68	1.07
Neurosurgery	66	1.04
Urology	65	1.02
Urology/Prostate	52	0.82
Trauma	46	0.73
Transplant	40	0.63
Family Practice	25	0.39
Orthopedics	25	0.39
Burn	16	0.25
Rehabilitation	14	0.22
Ear, Nose, Throat (Otolaryngology)	13	0.2
Ear, Nose, Throat	12	0.19
Dermatology	11	0.17
Dialysis	11	0.17
Long-Term Care	11	0.17
Long Term Care	7	0.11
Ophthalmology	7	0.11
Psychiatry	4	0.06
Critical Care Unit	3	0.05

Collectively, the geographic and demographic distributions observed in this surveillance data set primarily reflect patterns of healthcare-associated sampling and surveillance participation. These findings provide essential contextual information for interpreting subsequent susceptibility analyses, while underscoring the importance of sustained and geographically balanced surveillance to ensure representativeness over time.

### Antifungal susceptibility profiles and MIC shifts

**Fig 2:**
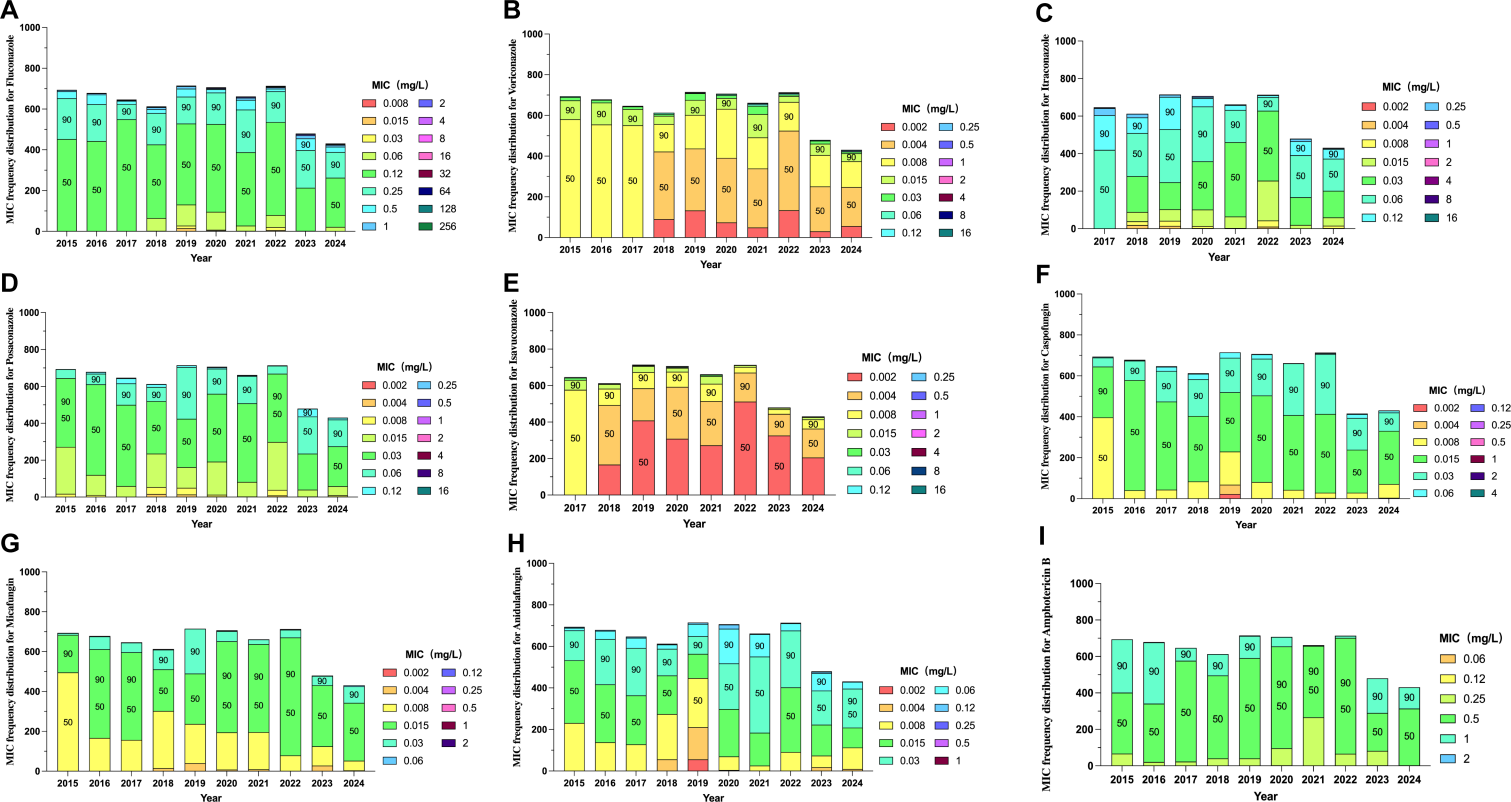
Antifungal minimum inhibitory concentration (MIC) distributions for *Candida albicans* isolates (2015–2024). MIC frequency distributions for triazoles (**A–E**), echinocandins (**F–H**), and amphotericin B (**I**) across annual isolate collections. Each panel illustrates temporal variation in MIC ranges and percentile thresholds (MIC_50_ and MIC_90_), depicting longitudinal trends in antifungal susceptibility among *C. albicans* isolates.

Across 6,342 *Candida albicans* isolates collected between 2015 and 2024, the MIC data revealed overall sustained susceptibility to echinocandins and amphotericin B, alongside early signals of azole MIC elevation. For fluconazole, MIC_50_ and MIC_90_ values were largely stable (0.12 and 0.25 mg/L) from 2015 to 2021 but showed a modest rise to 0.25–0.5 mg/L in 2023–2024 (Fig. 2A). Importantly, these changes occurred predominantly within the wild-type MIC distribution and remained below CLSI ECVs, indicating distribution-level variability rather than categorical resistance. The overall MIC range broadened over time (maximum MIC up to 256 mg/L), while the majority of isolates continued to cluster within low MIC ranges.

Voriconazole maintained consistently low MIC_50_ (0.004–0.008 mg/L) and MIC_90_ (0.008–0.015 mg/L) values throughout the decade (Fig. 2B). Although isolated higher MIC values (up to 16 mg/L) were observed in selected years (2022 and 2024), these represented infrequent events and did not alter the overall distribution pattern. Itraconazole and posaconazole exhibited overall stable activity (MIC_90_ ≤ 0.06 mg/L), yet both showed slight variability after 2022 (Fig. 2C and D). Isavuconazole demonstrated the narrowest distribution (MIC_90_ ≤ 0.008 mg/L) across all study years, indicating consistently preserved potency (Fig. 2E).

Among echinocandins, caspofungin, micafungin, and anidulafungin retained uniformly low MIC_50_ and MIC_90_ values (0.015–0.03 mg/L) without temporal shifts (Fig. 2F through H). Amphotericin B exhibited the most stable profile, with unchanged MIC_50_/MIC_90_ values (0.5/1 mg/L) throughout the surveillance period (Fig. 2I).

Taken together, these findings indicate sustained antifungal activity across major drug classes, with only low-level, distribution-based variability observed among azole MICs. Given the absence of formal statistical trend testing and the inherent variability of broth microdilution assays, these year-to-year changes should be interpreted as descriptive fluctuations within preserved susceptibility ranges, rather than evidence of progressive or clinically meaningful resistance development.

### Geographic variability in resistance patterns

**Fig 3:**
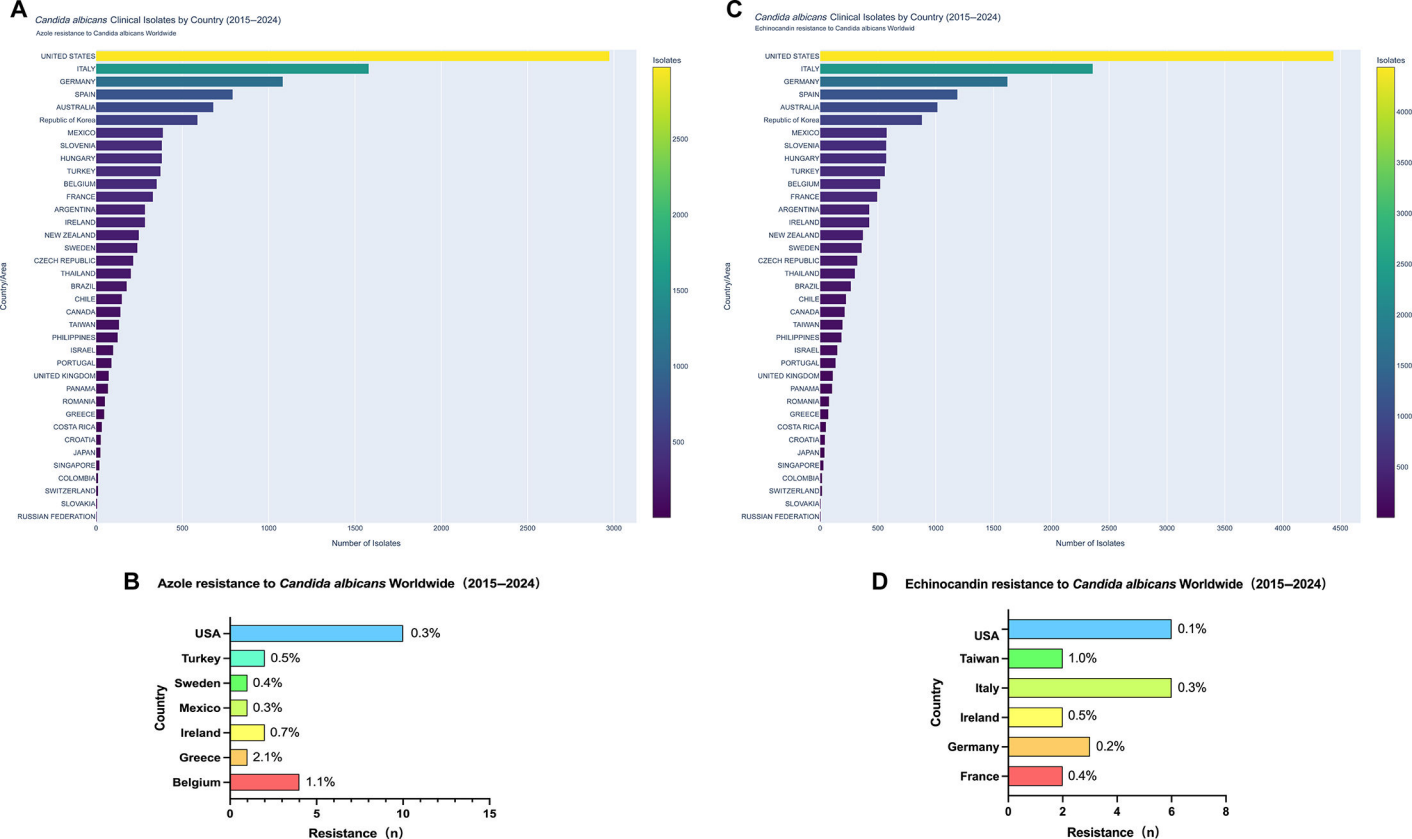
Geographic distribution and resistance frequencies of *Candida albicans* isolates (2015–2024). Country-level isolate counts for azole (**A**) and echinocandin (**C**) testing, and corresponding resistance frequencies (**B and D**). The figure illustrates geographic variability in antifungal susceptibility profiles across participating surveillance sites.

Despite extensive global sampling, resistance to both azoles and echinocandins among *Candida albicans* isolates remained reassuringly low. Across 41 surveyed countries between 2015 and 2024, azole resistance was detected in fewer than 1% of isolates in most regions. The highest resistance rates were reported in Greece (2.1%, *n* = 1), Belgium (1.1%, *n* = 4), and Ireland (0.7%, *n* = 2), followed by sporadic occurrences in Turkey (0.5%, *n* = 2) and the United States (0.3%, *n* = 10) (Fig. 3A and B). Echinocandin resistance was exceedingly rare, with overall rates below 1%, and was detected sporadically in Taiwan (1.0%, *n* = 2), Ireland (0.5%, *n* = 2), France (0.4%, *n* = 2), Italy (0.3%, *n* = 6), Germany (0.2%, *n* = 3), and the United States (0.1%, *n* = 6) (Fig. 3C and D).

These geographic patterns illustrate localized variability in resistance frequency within an overall context of low global prevalence. Given the limited absolute numbers of resistant isolates in individual countries, these findings should be interpreted cautiously and primarily as descriptive observations highlighting the value of region-specific surveillance rather than evidence of widespread dissemination.

### Temporal dynamics of azole and echinocandin resistance

**Fig 4:**
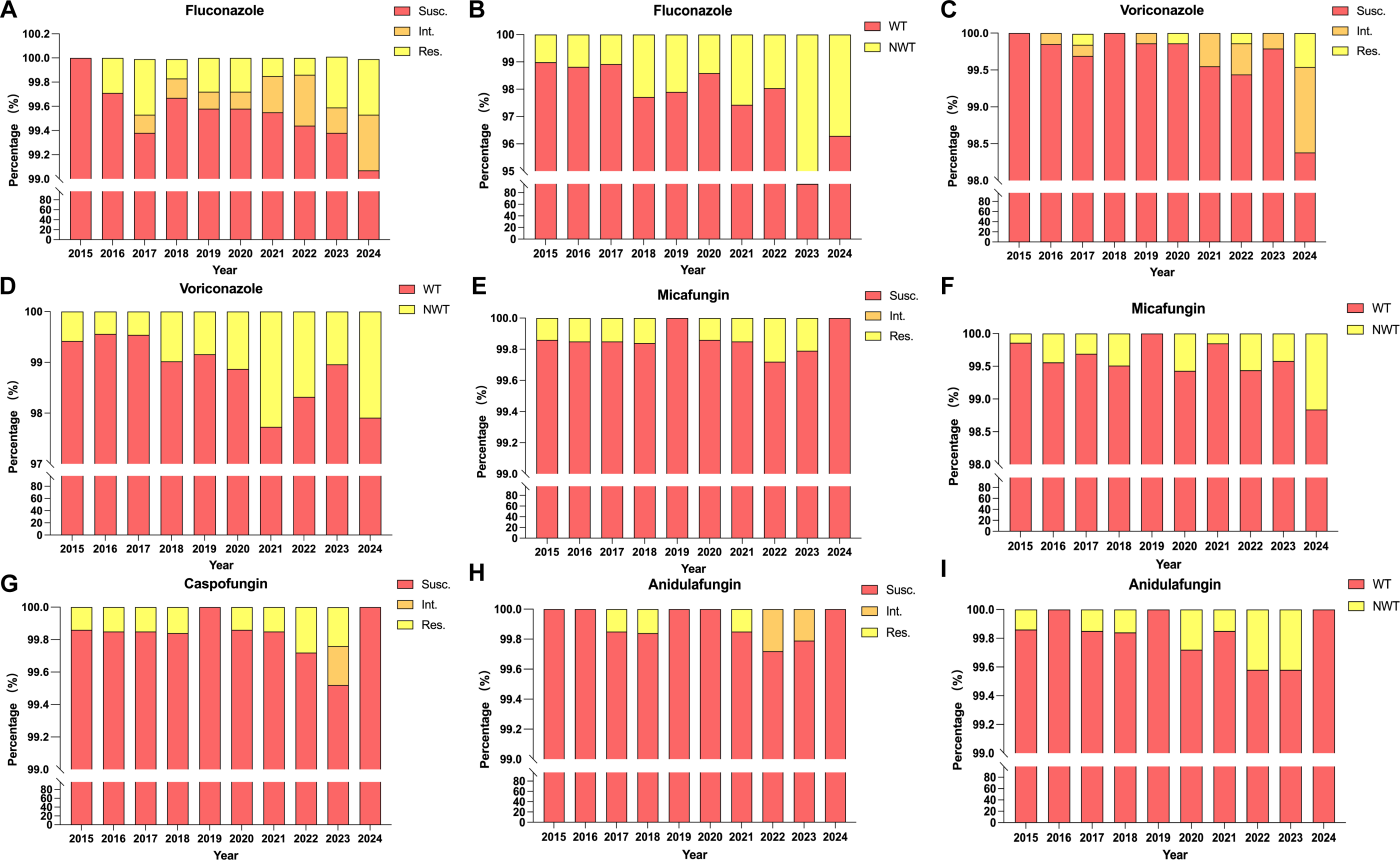
Temporal trends in antifungal susceptibility of *Candida albicans* (2015–2024). Yearly susceptibility and wild-type/non–wild-type (WT/NWT) proportions for azoles (**A–D**) and echinocandins (**E–I**). The figure illustrates sustained echinocandin activity and low-magnitude variability in azole susceptibility over the surveillance period.

Over the decade-long surveillance period (2015–2024), *C. albicans* susceptibility to both azoles and echinocandins remained high, though subtle temporal patterns emerged. Fluconazole susceptibility remained consistently above 97%, with annual values ranging from 99.7% in 2016 to 99.1% in 2024 (Fig. 4A). Correspondingly, the proportion of NWT isolates, as defined by CLSI ECVs, increased from 1.0% in 2015 to 3.7% in 2024 (Fig. 4B). These changes occurred gradually and remained within low absolute frequencies, without abrupt shifts in susceptibility categories. Voriconazole displayed a similar pattern, with susceptibility remaining above 98% across all years (Fig. 4C). The proportion of NWT isolates increased from 0.6% in 2015 to 2.1% in 2024 (Fig. 4D), reflecting distribution-level variability rather than a transition to widespread resistance. For both azoles, observed changes were characterized by small absolute differences and should be interpreted in the context of preserved overall susceptibility and assay-level variability.

In contrast, echinocandin agents (caspofungin, micafungin, and anidulafungin) maintained exceptionally stable activity. For micafungin and caspofungin, susceptibility rates consistently exceeded 99.7%, and no resistant isolates were observed throughout the study period. The proportion of NWT isolates fluctuated minimally between 0.1% and 0.6%, without evidence of progressive accumulation (Fig. 4E through G). Similarly, anidulafungin retained >99.5% susceptibility with transient (Fig. 4H), sporadic NWT detection (<0.4%) (Fig. 4I), indicating no significant drift in echinocandin MIC distributions over time.

Collectively, these temporal data demonstrate stability of echinocandin susceptibility and high overall azole activity, accompanied by low-level, descriptive variability in WT/NWT proportions over time. In the absence of formal statistical trend testing, these observations should be interpreted as distributional fluctuations within preserved susceptibility ranges, underscoring the value of longitudinal surveillance for contextualizing subtle changes rather than indicating the development of progressive resistance.

## DISCUSSION

Invasive candidiasis continues to pose a substantial threat to hospitalized and immunocompromised populations, with *Candida albicans* remaining one of the most common etiologic agents in long-running global surveillance programs (1, 12). Our analysis of more than 6,300 *C. albicans* isolates from the ATLAS program represents a large, decade-scale, single-species, globally standardized evaluation of antifungal susceptibility. The data reaffirm the sustained potency of echinocandins and amphotericin B—supporting their continued use as reliable agents in empirical therapy—while also revealing low-magnitude variability in azole susceptibility and geographically localized differences in resistance frequency. These findings highlight the necessity of reinforcing antifungal stewardship and tailoring empirical regimens to local epidemiological patterns. Furthermore, they underscore the importance of integrating molecular surveillance and pharmacodynamic modeling to better contextualize observed susceptibility patterns over time.

Large-scale surveillance initiatives, most notably the SENTRY Antifungal Surveillance Program, have provided an essential foundation for understanding global trends in *Candida* epidemiology and antifungal resistance over extended periods (13, 16–21). The landmark analysis by Pfaller et al. (22), encompassing more than 20,000 invasive *Candida* isolates across multiple species and two decades, demonstrated that resistance among *C. albicans* remained uncommon worldwide, particularly to echinocandins and amphotericin B, while documenting slow, species-specific shifts in azole susceptibility. Our findings are largely concordant with these observations, confirming that *C. albicans* has not undergone widespread shifts toward clinically significant resistance. By contrast to multi-species surveillance designs, the present study focuses exclusively on *C. albicans* and applies a temporally dense, decade-long framework (2015–2024), allowing closer examination of within-species MIC distributions over time. Within this context, we observed modest upward shifts in fluconazole and voriconazole MIC distributions after 2022, changes that remain within low absolute resistance frequencies but may be less apparent in broader, species-aggregated analyses. In addition, the identification of geographically confined differences in resistance frequency—such as higher azole resistance in Greece and sporadic echinocandin resistance in select regions—suggests that susceptibility variability in *C. albicans* is more likely to emerge in localized settings, consistent with previous genomic studies describing regionally restricted resistant subclades rather than global dissemination (23, 24).

From a clinical standpoint, our findings provide important contextual information for antifungal therapy and stewardship. The persistently high susceptibility to echinocandins supports their continued use as highly reliable first-line agents for invasive *C. albicans* infections, particularly in critically ill or elderly patients, where delays in effective treatment are linked to increased mortality. Amphotericin B, though limited by toxicity, remains an indispensable alternative in settings of echinocandin intolerance or restricted access. At the same time, azole susceptibility remained high across regions, and the observed low-magnitude MIC variability and regional differences support a measured and evidence-based approach to azole use, emphasizing their role in step-down or targeted therapy based on confirmed susceptibility results. The emergence of geographically confined resistance foci further emphasizes the need for regionally adaptive treatment guidelines and dynamic stewardship frameworks that integrate both phenotypic and molecular data. Continuous, geographically resolved surveillance will be essential to support informed therapeutic decision-making in an evolving resistance landscape.

Looking forward, these findings highlight several areas where further investigation would enhance interpretation of long-term surveillance data. Molecular studies may help clarify the mechanisms underlying observed variability in azole susceptibility, including the contributions of *ERG11* alterations, efflux regulation, and stress-response–associated adaptive tolerance. Although echinocandin resistance remains uncommon in *C. albicans*, previous studies have shown that reduced susceptibility can arise through chromosomal aneuploidy or mutations in *FKS1* and *FKS2* affecting β-(1,3)-D-glucan synthase activity (25, 26), underscoring the value of continued mechanistic investigation. Future work may also benefit from integrating molecular epidemiology with pharmacodynamic and clinical outcome data to refine interpretation of phenotypic susceptibility patterns. In parallel, continued development of antifungal agents with novel mechanisms of action remains important to sustain therapeutic options against *Candida* species. Together, such efforts would strengthen the interpretive framework linking surveillance data, biological mechanisms, and clinical decision-making.

Despite its scope and standardized design, this study has several limitations that warrant discussion. First, the ATLAS data set primarily captures hospital-based surveillance, likely overrepresenting tertiary-care and intensive care populations while underestimating community-acquired infections and outpatient settings. Second, detailed clinical metadata—including antifungal exposure history, infection versus colonization status, and patient outcomes—were not available, precluding direct assessment of the clinical impact of observed MIC variability or resistance frequency and limiting stewardship inferences to a descriptive context. Third, although all susceptibility testing was performed under centralized quality control using standardized CLSI methodologies, regional differences in sampling intensity and isolate contribution may have influenced geographic resistance estimates, particularly in settings with smaller denominators. Moreover, WT/NWT classification was based exclusively on ECVs, without molecular confirmation, and phenotypic non–wild-type status does not necessarily reflect established genetic resistance mechanisms. In addition, because broth microdilution testing is subject to intrinsic inter-assay variability (typically within one twofold dilution), the low-magnitude MIC shifts observed over time cannot be confidently distinguished from methodological variability. Finally, the reduced isolate numbers after 2022—possibly reflecting pandemic-related disruptions—may have modestly affected precision in temporal trend analysis. These limitations underscore that the findings should be interpreted as descriptive surveillance observations rather than causal or predictive assessments. Nonetheless, the large sample size, decade-long surveillance window, and globally standardized testing protocols support the robustness of the overall susceptibility patterns described.

In summary, *Candida albicans* remains predominantly susceptible to echinocandins and amphotericin B worldwide, with azole susceptibility also remaining high across regions. The low-magnitude variability observed in azole MIC distributions and the presence of geographically localized differences in resistance frequency should be interpreted within the context of biological and surveillance variability rather than as evidence of widespread resistance emergence. These findings reinforce the continued value of standardized, long-term global surveillance to contextualize subtle susceptibility changes, inform regionally appropriate antifungal stewardship, and support evidence-based clinical decision-making in the coming decade.

## Data Availability

All data supporting the findings of this study are openly available from the Pfizer ATLAS database at https://atlas-surveillance.com/, under an open-access license (CC-BY 4.0). The data that support the findings of this study can be accessed using the persistent identifier DOI https://doi.org/10.6084/m9.figshare.30372514.
